# Interactive Lane Keeping System for Autonomous Vehicles Using LSTM-RNN Considering Driving Environments

**DOI:** 10.3390/s22249889

**Published:** 2022-12-15

**Authors:** Yonghwan Jeong

**Affiliations:** Department of Mechanical and Automotive Engineering, Seoul National University of Science and Technology, 232 Gongneung-ro, Nowon-gu, Seoul 01811, Republic of Korea; yh.jeong@seoultech.ac.kr; Tel.: +82-2-970-6322

**Keywords:** autonomous vehicle, decision making, lane keeping, long short-term memory, machine learning, recurrent neural network

## Abstract

This paper presents an interactive lane keeping model for an advanced driver assistant system and autonomous vehicle. The proposed model considers not only the lane markers but also the interaction with surrounding vehicles in determining steering inputs. The proposed algorithm is designed based on the Recurrent Neural Network (RNN) with long short-term memory cells, which are configured by the collected driving data. A data collection vehicle is equipped with a front camera, LiDAR, and DGPS. The input features of the RNN consist of lane information, surrounding targets, and ego vehicle states. The output feature is the steering wheel angle to keep the lane. The proposed algorithm is evaluated through similarity analysis and a case study with driving data. The proposed algorithm shows accurate results compared to the conventional algorithm, which only considers the lane markers. In addition, the proposed algorithm effectively responds to the surrounding targets by considering the interaction with the ego vehicle.

## 1. Introduction

The advancement of an active safety system improves road safety by preventing accidents caused by driver carelessness. At the beginning of active safety, a camera sensor is used to provide input for the Lane Departure Warning System (LDWS) [1]. The information from the various environment sensors is used to implement the Forward Collision Warning System (FCWS) [2]. A corner radar and ultrasonic sensor are used for Blind Spot Warning (BSW). Based on the success of the active safety system, the Advanced Driver Assistant System (ADAS) was introduced to provide convenience [3]. The functions of the ADAS can be classified into two categories, parking and driving assistance. The parking assist system provides steering control for parallel and vertical parking using an ultrasonic sensor [4]. Rear Autonomous Emergency Braking (AEB) provides emergency braking in a case where there is a risk of collision between surrounding objects and the vehicle moving backward [5]. The driving assist functions are classified into two categories, longitudinal and lateral controls. AEB was developed to mitigate or avoid collision with a front target, such as a vehicle, pedestrian, or cyclist [6]. To improve convenience, adaptive cruise control was developed to decide the desired longitudinal acceleration to maintain safety clearance with a preceding target [7,8]. For lateral control, the Lane Keeping Assist System (LKAS) overlays an assist torque on the steering system to prevent lane departure due to the driver’s careless driving [9]. So, an early version of the LKAS only assists when the vehicle is about to depart the lane. Recently, highway driving assist is introduced by integrating the ADAS functions for longitudinal and lateral controls [10]. The success of the ADAS led to research on autonomous driving. The ADAS has provided many conveniences and contributed to improving road safety. However, many drivers feel discomfort due to the different driving patterns of the ADAS. In particular, more issues have been raised for the lateral functions, because the control margin is quietly smaller than the longitudinal one [11,12].

Many kinds of research have been conducted to develop the LKAS and Lane Following Assist (LFA) algorithm to improve the lane keeping performance. For the convenience of description, LKAS and LFA are collectively referred to as LKAS. The methodology for LKAS can be classified into model-based and learning-based methods. For a model-based controller, a PID controller was used to determine the steering input based on the error between the vehicle yaw rate and reference [13,14]. A fuzzy logic-based algorithm was designed to model the nonlinear steering response to the lateral offset [15,16]. The Takagi–Sugeno–Kang (TSK) fuzzy extension controller was utilized to improve the control performance when switching controllers [16]. To manage the control authority problem, shared control was proposed [17,18,19]. In addition, mode switching strategy was utilized for the transition between LKAS and waypoint tracking [20]. An adaptive controller was used to configure a self-tuning regulator for LKAS [21]. In addition, a Sliding Mode Control (SMC) was applied to LKAS. Quasi-continuous SMC was introduced to reduce lateral and heading errors with the driving lane [22,23,24]. To find the optimal control inputs, Model Predictive Control (MPC) was utilized [25,26,27]. A potential field was introduced to provide the steering assist without tracking the desired path [28].

The model-based approach requires a precise model to achieve the LKAS performance. Learning-based approaches have been applied to design the LKAS algorithm to overcome the modeling error. Initially, a shared steering controller was designed by using online learning [29]. To improve the LKAS intervention timing, a Gaussian Mixture Model (GMM) was trained to establish the relationships between the variables related to lane keeping. A Hidden Markov Model (HMM) used GMM to estimate the future lateral position of the vehicle to warn of lane departure [30]. HMM and Gaussian Mixture Regression (GMR) provided the most likely inputs of the driver, which were used as the reference state for MPC [31] Since the LKAS relies on lane measurement from the front camera, an end-to-end approach is utilized to decide the steering input directly from the image [32,33,34,35,36,37]. Since the behavior of the vehicle is governed by the dynamic equation, a Recurrent Neural Network (RNN) was introduced to learn the temporal dependency of the LKAS [34]. To improve the performance of lane keeping, the driving data were classified into three categories, going straight, turning right, and turning left. Convolutional Neural Networks (CNN) were generally used to generate the road model for LKAS from the image [38]. In addition, the steering angle can be determined directly using a CNN. The three 3D CNN with Long Short-Term Memory (LSTM) were trained for each data to realize the end-to-end LKAS method [35]. Similarly, a CNN-based end-to-end LKAS algorithm was proposed to directly use the image for steering angle decisions [36]. Imitation learning was introduced to train the policy based on CNN [37].

To overcome the difficulty in collecting and labeling the training data samples, Reinforcement Learning (RL) is utilized to determine the steering angle from interaction with driving environments. Q-learning was used to design the end-to-end LKAS without a model. The reward function was designed to regulate the lateral and heading deviations from the road center [39]. The integration of the model-free RL and direct yaw moment control was proposed to negotiate lane keeping and lateral stability [40]. To enhance the performance of the Q-learning, Deep Q-Network (DQN) with discrete action space was used to improve the Q-function by introducing a deep neural network [41]. Since the steering input is continuous, it is necessary to perform RL based on the continuous action space. Therefore, Deep Deterministic Policy Gradient (DDPG) was used to design reinforcement learning-based LKAS [41,42]. Similarly, the deep deterministic actor–critic algorithm is used to configure the LKAS [32]. Since the LKAS only relied on the camera, the road over the detection range can be considered uncertain. To be aware of the uncertainty, the convolutional mixture density network was designed to estimate the future lateral and heading error [43]. For the performance enhancement of RL, a Monte Carlo tree search was applied to improve the convergence of RL [44].

**Figure 1:**
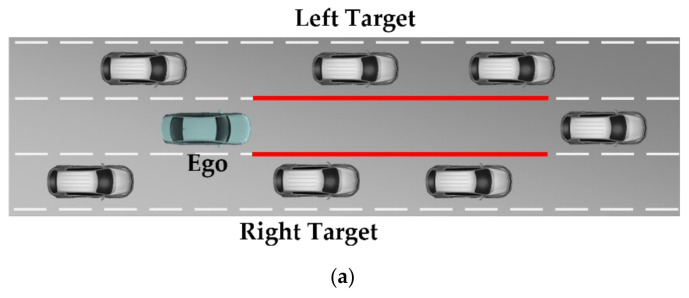

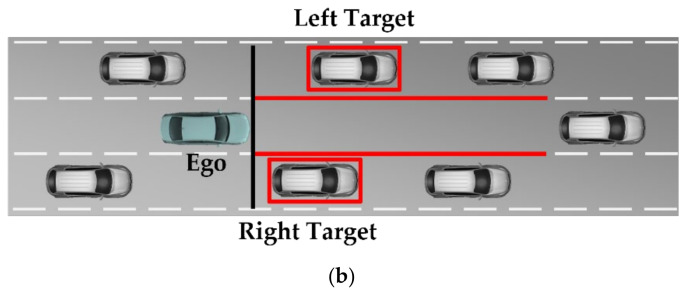
Concept of the lane keeping assistant system: (**a**) conventional lane keeping assistant system and (**b**) interactive lane keeping assistant system.

From a careful review of the previous studies, various kinds of methods have been introduced to design the LKAS. The early LKAS had an issue regarding switching control with drivers. Therefore, studies on shared control were conducted. Then, the operating conditions of the LKAS were extended from lane departure prevention to actively following the center of the road. To improve the performance of the LKAS, a robust controller is used to achieve the lane keeping performance in various driving conditions. Since the steering system and lateral vehicle dynamics are nonlinear, adaptive control and SMC are introduced to design the LKAS algorithm. Recently, learning-based approaches were introduced to overcome the limitations of the model-based approach. However, the research on LKAS focused on lane keeping only considering the shape of the lane and road. This means that the effect of the surrounding vehicles is not reflected in the steering angle decision. Figure 1 shows the concept of the conventional and proposed LKAS. As shown in Figure 1a, the conventional approaches determine the steering angle based on the detected lane markers. However, the driver considers the surrounding targets when determining the steering input for lane keeping. Therefore, LKAS should consider the surrounding targets to improve driver acceptance and lane keeping performance.

The contributions of this paper are summarized as follows:

1. The proposed algorithm reflects the driver’s consideration for the surrounding targets when determining the steering wheel angle input to follow the lane.
2. The proposed algorithm is designed in consideration for changes in the length of the input data so that it can respond to changes in the number of surrounding vehicles.
3. Information on surrounding vehicles was accumulated with lane markers for a specific time and used as input to consider the interaction between vehicles.

This study presents an interactive LKAS for the Autonomous Vehicle (AV) with a learning-based approach, which uses RNN with LSTM cells. The goal of this study is to improve the driver’s acceptance of the lane keeping by learning the driver’s characteristics. Thus, the proposed LKAS is designed to reflect the interaction between the AV and the surrounding vehicles. As shown in Figure 1b, the nearest target on the front left and right, which are highlighted as red boxes, are considered with lane information. To train the proposed interactive LKAS algorithm, driving data were collected by a Data Collection Vehicle (DCV). Three drivers drove the DCV on a highway in South Korea to avoid overfitting the driving pattern of the specific driver. The driving data-based analysis and case study is conducted to evaluate the effectiveness of the proposed algorithm. 

## 2. Data Collection

The learning-based approach requires quality data samples to achieve the desired performance. In many studies, open datasets have been frequently used to provide the basis for the decision-making and motion planning for ADAS and AVs. Typical examples of an open dataset are NGSIM, KITTI, and Argoverse. Using open datasets has the advantage of saving time and cost for data collection and comparing them to other studies using the same data. However, if the data obtained from the target system are different from the open dataset, it is difficult to directly use the algorithm based on the open datasets. For example, NGSIM datasets were collected by a surveillance camera. Therefore, it contains a variety of data that are difficult to recognize with sensors mounted on the vehicle. In this study, driving data were collected by a DCV. It means that the algorithm using the collected data can be directly applied to the AVs. In other words, training and validation were conducted using measurable data from the sensors mounted on the AV. Since information, which is obtained from the environment and the chassis sensors, are synchronized and stored, the collected datasets include interaction between the AV and the surrounding targets. The details of the DCV, data collection road, and data sample generation are described in the following sections.

### 2.1. Vehicle Configuration

**Figure 2:**
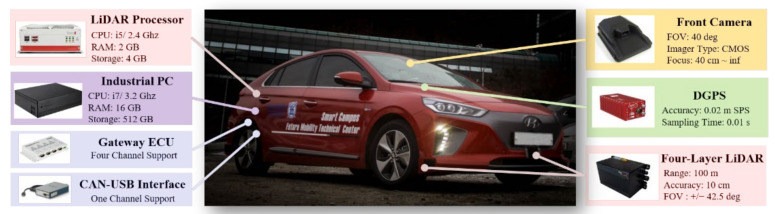
Configuration of the data collection vehicle. Adapted from ref. [45].

**Figure 3:**
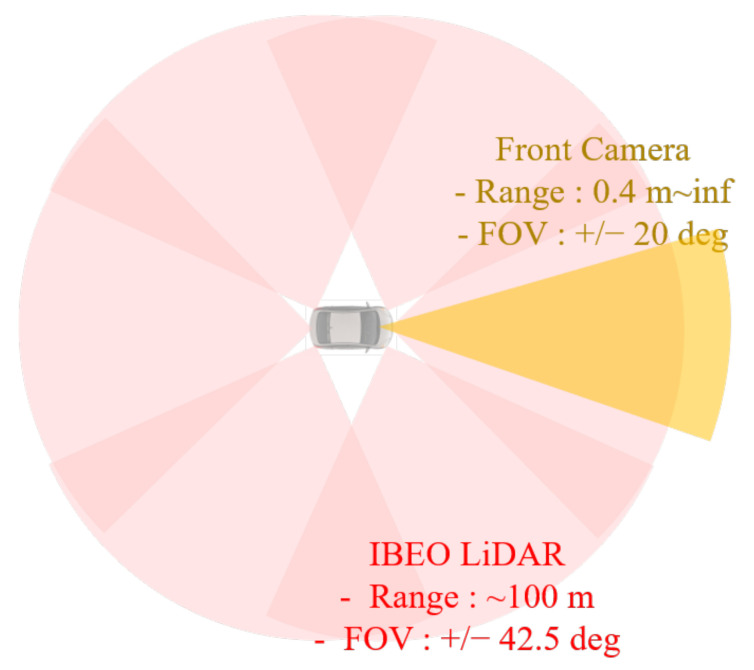
Field of view of sensors mounted on the data collection vehicle. Adapted from ref. [45].

DCV was designed for autonomous driving in various environments. The configuration of the DCV is shown in Figure 2. Six LiDAR and a dedicated processor are used to detect the objects around the DCV. In this study, IBEO LUX and HAD Feature Fusion System are utilized to achieve all-around object detection in the Local Coordinate System (LCS) of the DCV. The object information includes relative position, heading, and velocity in LCS. The origin of the LCS is located at the center of the rear axle. The LCS, which is the right-handed coordinate system, has the x-axis in the direction where the DCV travels. In addition, the object information includes the class of the target. Considering the vehicle, the class is composed of passenger cars, heavy vehicles, and unknown moving objects. In this study, the objects classified as passenger cars and heavy vehicles are used to define the targets in the left and right lanes, which are shown in Figure 1. The front camera is used to detect the lane markers. Mobileye Q3 is adopted as the front camera. This camera module provides the shape of the lane markers as a second-order polynomial with detection ranges. In addition, the quality of lane detection is evaluated from zero to three. Since the front camera can detect the vehicle, the object information from the front camera is also used to supplement an object classification by fusing with the LiDAR detection results. The Field of View (FOV) of the DCV is described in Figure 3. As shown in Figure 3, the environment sensors cover the all-around FOV around the DCV. The chassis sensors are used to measure the dynamic states of the DCV. The chassis sensors for Steering Wheel Angle (SWA), wheel speed, and yaw rate are used to measure the dynamic states of the DCV. To accumulate the data in the global coordinate system, a Differential Global Positioning System (DGPS) is used. OxTS RT3002 is used as the DGPS for the DCV, which measures the global position with a Circular Error Probability (CEP) of 0.02 m. The measured longitude and latitude are converted to the Universal Transverse Mercator (UTM) coordinate system.

Additional equipment is used to collect and save the data from the sensors. All data from each sensor are collected and stored by an industrial PC. Therefore, each datum is synchronized. In addition, a global timestamp is assigned for each sampling time using the time information of the DGPS. The global timestamp is used to reconstruct the driving data for data sample generation. The interface between the PC and Controller Area Network (CAN) is built using a CAN-USB interface device. A gateway Electronic Control Unit (ECU) selects only the corresponding information in the chassis CAN and outputs it through a separate CAN, which is connected to the CAN-USB interface device.

### 2.2. Data Collection Road

**Figure 4:**
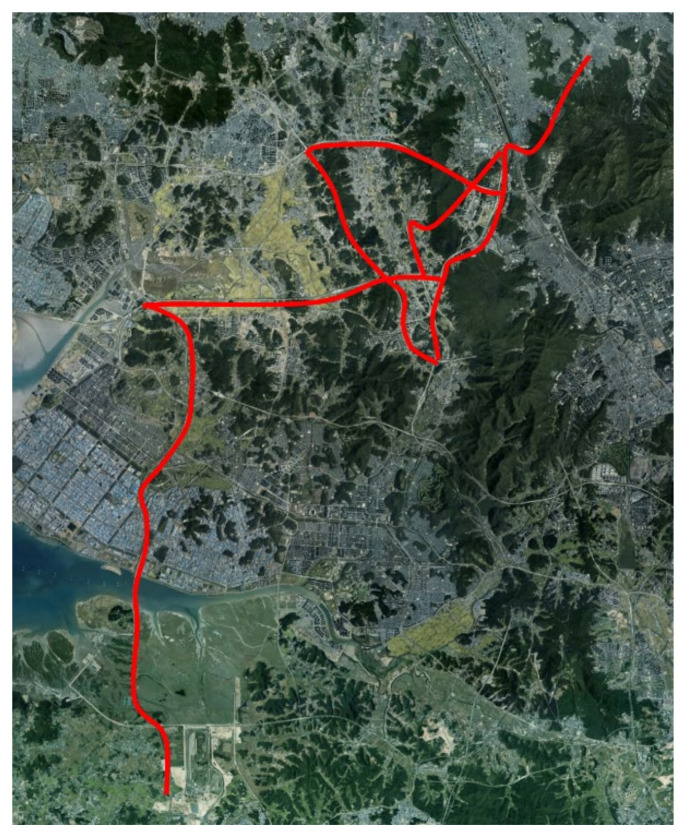
Data collection road indicated on a satellite map.

**Figure 5:**
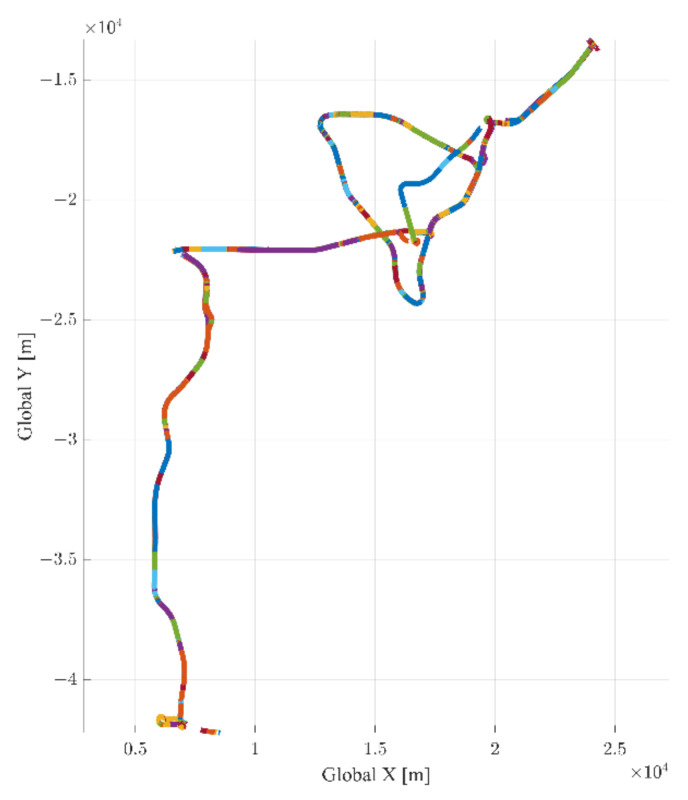
Collected driving trajectory in the UTM coordinate system.

The driving data are collected by driving on various highways in South Korea. Figure 4 shows the data collection road, which is indicated on a satellite map. To acquire the various lane keeping driving data, the data collection road is composed of the following six expressways: Seohaean, Second Gyeongin, Third Gyeongin, Pyeongtaek Siheung, capital region first ring, and Gangnamsunhwan-ro. Therefore, the data collection road covers a various number of lanes and a range of traffic. For example, the capital region first ring expressway consists of four lanes in one way and passes through major residential cities of Gyeonggi-do, South Korea. Therefore, this expressway has a lot of traffic and a high proportion of passenger cars. In contrast, Pyeongtaek Siheung expressway is composed of two or three lanes in one direction and connects industrial areas. Therefore, the proportion of heavy vehicles is relatively higher than other expressways. Figure 5 shows the example trajectories of the DCV in the UTM coordinate system with different colors.

### 2.3. Data Sample Generation

**Figure 6:**
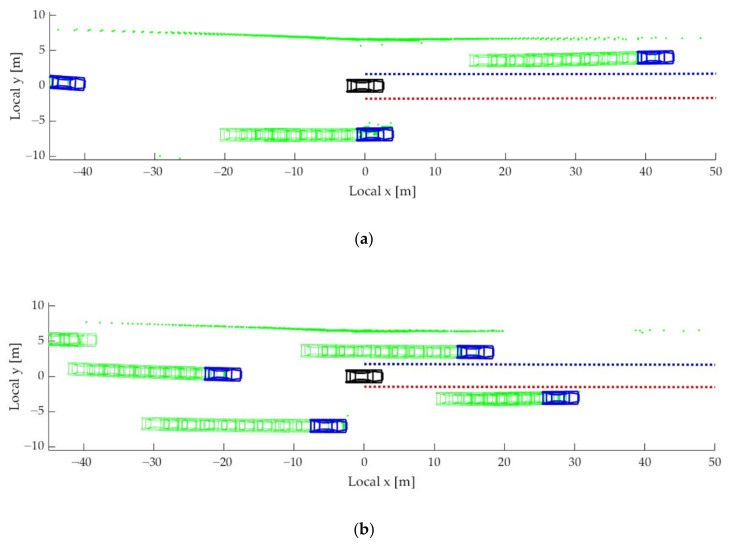

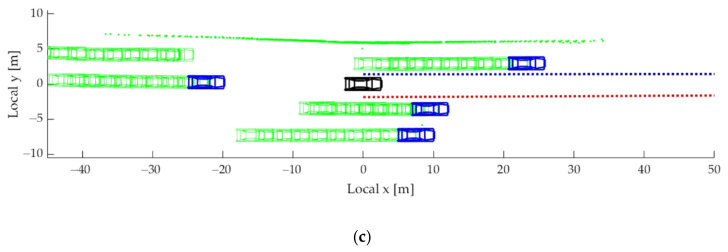
Example of the input sequences for training, validation, and testing: (**a**) a case of not being affected by the surrounding vehicle; (**b**) a case of being affected by the vehicle in the left lane; and (**c**) a case of being affected by the vehicle in the right lane.

**Figure 7:**
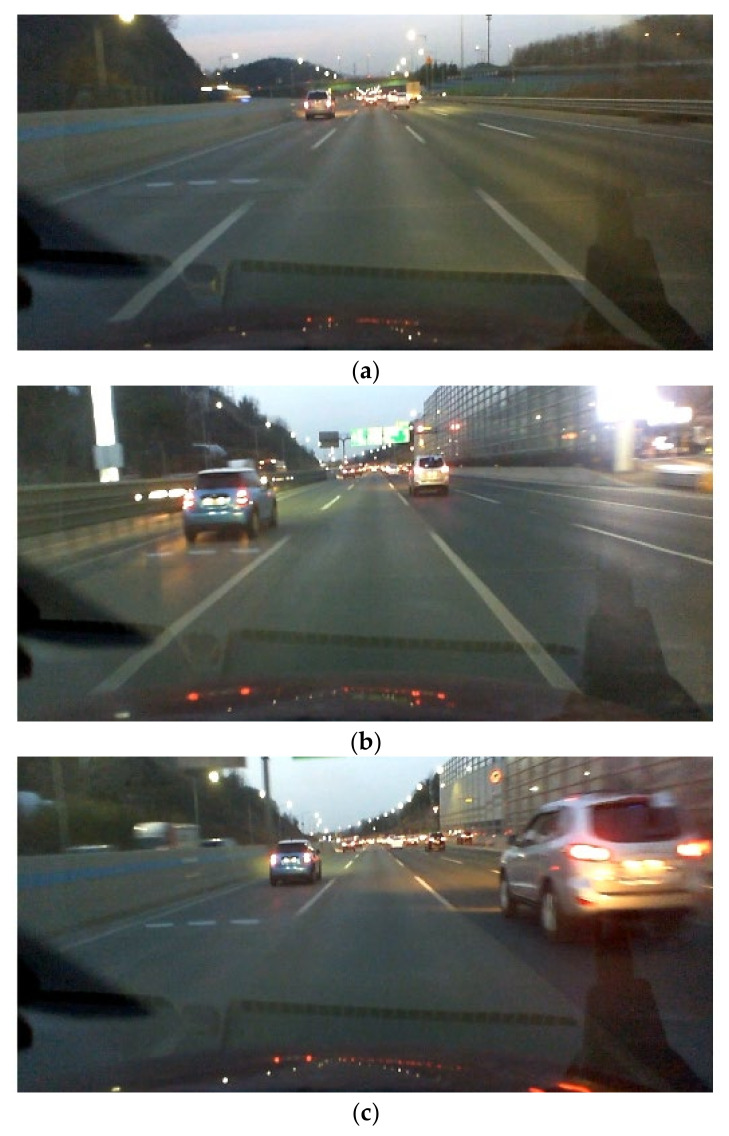
Dashcam logs: (**a**) a case of not being affected by the surrounding vehicle; (**b**) a case of being affected by the vehicle in the left lane; and (**c**) a case of being affected by the vehicle in the right lane.

The collected driving data should be processed to generate the training, validation, and test data samples. Each data sample is composed of input and output sequences. Lane markers and the position of the target vehicles are transformed into UTM coordinate system by using the position information acquired by DGPS. This allows for generating input and output sequences in a continuous trajectory at a specific moment. As mentioned in the introduction, the proposed interactive LKAS algorithm considers not only the lane makers but also the surrounding targets when determining the SWA. Figure 6 shows examples of the input sequences, which are reconstructed from the collected driving data. Figure 7 shows the dashcam logs at the same time as Figure 6. In Figure 6, the DCV and the surrounding targets are depicted as black and blue vehicles, respectively. The dotted blue and red lines represent the left and right-lane markers. The accumulated histories of the surrounding targets are marked as green vehicles. The point clouds from the LiDAR are depicted as green dots. Since the points from the ground are rejected by the perception algorithm, only points from vehicles and guardrails are marked.

The collected driving data are composed of 116 logs, each log is on average 5 min long. Therefore, the collected data total is about 580 min long. In this study, lane detection quality is used to select the appropriate data to generate the data samples. Data with the lane detection quality for both lanes falling below 3 were excluded. This is because the LKAS system can be activated when one of both lane markers is normally detected. After processing the collected driving data, 46,355 data samples were generated. These data samples were divided into 70% for training, 20% for validation, and 10% for testing. Therefore, 32,449; 9281; and 4635 data samples are used for training, validation, and testing for the proposed interactive LKAS algorithm.

## 3. Driving Characteristics Analysis

The objective of the proposed algorithm is to reflect the lane keeping characteristics in the LKAS when there are surrounding vehicles. In other words, the proposed LKAS considers not only the lane measurement but also the left- and right-lane targets. Figure 6 also shows the driving characteristics of the driver who considers the surrounding vehicles in lane keeping situations. As shown in Figure 6a, the driver followed the center of the lane because there is no adjacent target around the DCV. In contrast, in the case where a vehicle on the left or right lane was close to the DCV, the driver tried to reduce the risk by following the lane biased in the opposite direction. For example, as shown in Figure 6b, the DCV was biased to the right-lane marker to secure a sufficient level of lateral clearance with the left-lane target. Similarly, the driver drove the vehicle close to the left-lane marker to increase the lateral clearance with the right-lane target, as shown in Figure 6c.

**Figure 8:**
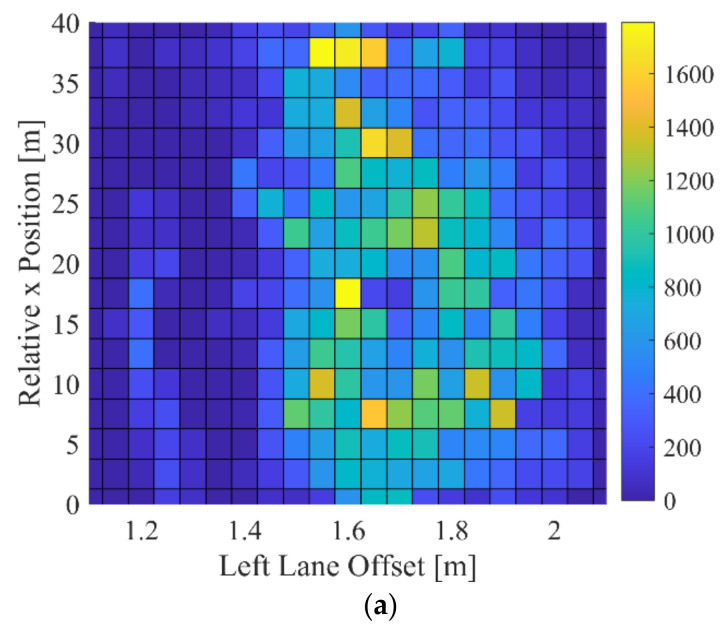

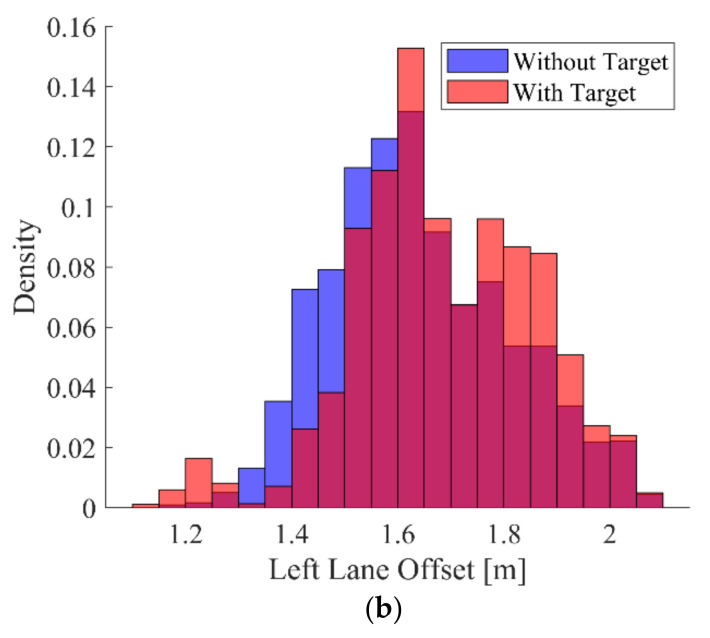
Lane keeping characteristics when left-lane target exists: (**a**) distribution of left-lane offset and relative x position when left-lane target exists and (**b**) histogram of left-lane offset depending on the presence of left-lane target.

**Figure 9:**
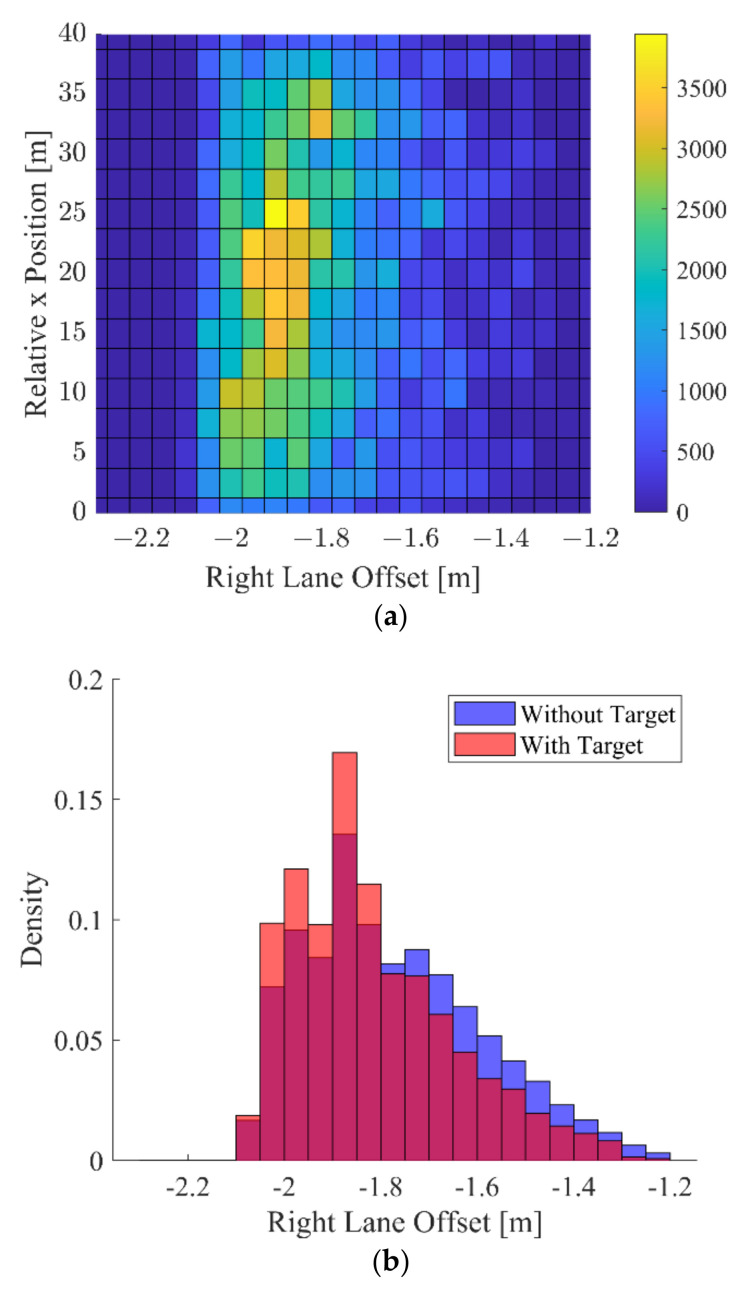
Lane keeping characteristics when right-lane target exists: (**a**) distribution of right-lane offset and relative x position when right-lane target exists and (**b**) histogram of right-lane offset depending on the presence of right-lane target.

To analyze the driver’s consideration of the surrounding vehicles when lane keeping, the collected driving data were divided into three cases, the case with left-lane target, right-lane target, and without any target. First, the distribution of the relative x position of the left-lane target and left-lane offset of the DCV is depicted in Figure 8a. As shown in Figure 8a, the driver tried to maintain the left-lane offset as 1.6 m when the left-lane target was far enough away. In contrast, as the left-lane vehicle approaches the DCV, the distribution of the left-lane offset gradually widens to a value larger than 1.6 m. A histogram of the left-lane offset is shown in Figure 8b. When there is a left-lane target, it can be seen that the distribution of around 1.8 m increased compared to the case without a target. In contrast, the distribution around 1.4 m decreased. A similar phenomenon is shown when a right-lane target exists. As shown in Figure 9, the distribution of the right-lane offset is shifted farther away from the right-lane target. In South Korea, the driver’s seat is on the left. This means that there are more blind spots on the right side of the vehicle. The distribution of right-lane offset is biased toward keeping a distance more than the case of left-lane offset. Since the right side has more lateral clearance to the right-lane marker even in normal driving, the effect of the surrounding vehicles is greater than the case of the left-lane target.

## 4. Interactive LKAS Algorithm

This study proposed the interactive LKAS algorithm based on the LSTM-RNN. Since the interactive LKAS algorithm considers the lane shape and the surrounding targets, appropriate input features and preprocessing should be defined first. If an appropriate input feature is not selected, the neural network cannot sufficiently reflect the driver’s driving characteristics. In addition, an excessive number of input features increase the computational burden, making it difficult to use in real-time applications. Furthermore, the scale of each input feature is quietly different because the proposed algorithm uses the information from the various sensors. Therefore, it is necessary to adjust the range of each feature similarly in order to improve learning performance.

### 4.1. Features and Preprocessing

The first step of the learning-based approach is defining the input and output features of the neural network. Since the goal of this study is to design the LKAS algorithm, the output feature is the SWA. However, determining the input feature requires consideration of various information such as the state of vehicles and lane markers. For vehicle ego states, SWA, steering angle speed, longitudinal velocity, yaw rate, and longitudinal and lateral accelerations are the input feature candidates. Among this information, steering angle speed and acceleration are excluded due to measurement noise. Therefore, SWA, longitudinal velocity, and yaw rate were used as input features to express the state of the ego vehicle. For surrounding vehicles, the x-position, y-position, heading angle, and speed of the surrounding vehicles with respect to the LCS are used as input features. In the case of lane information, lane markers were used as input features. The camera provides the lane marker in the form of second-order polynomials. In other words, lateral offset, heading angle, and curvature of each lane maker are considered.

There is a large difference in the scale of the input features. For example, the scale of the longitudinal velocity ranges from 10 to 10^2^, and the curvature is about 10^−4^. If a neural network is trained with data composed of features on different scales, features with small scales are not properly learned. In this study, if the collected driving data is learned as it is, the curvature, heading angle, and yaw rate will be ignored. In this study, standardization is used to convert the mean and standard deviation of each input feature to zero and one. The standardization is performed as follows:

(1) $${\bar{x}}_{k,n}=\frac{x_{k,n}-\mu_{n}}{\sigma_{n}}$$

where *x_k_*_,_*_n_* is the *n*-th input feature at *k*-th time stamp, and *μ_n_* is the mean and *σ_n_* is the standard deviation of the *n*-th input feature. In this study, the collected driving data were classified into three categories: training, validation, and testing. *μ_n_* and *σ_n_* are derived from the training data samples. These *μ_n_* and *σ_n_* are saved and reused to process the validation and test data samples. Since the neural network is trained by standardized data samples, the output of the neural network is converted to physical quantities as follows:

(2) $$x_{k,n}=\sigma_{n}\cdot {\bar{x}}_{k,n}+\mu_{n}$$

The same *μ_n_* and *σ_n_* in (1) are used to convert the output of the neural network to physical scale values.

### 4.2. Neural Network Design

The lane keeping characteristics of the driver has a temporal dependency. For example, if the driver observes the left-lane target approaching the ego vehicle, the driver tries to move closer to the right-lane marker to secure sufficient lateral clearance with the left-lane target. In addition, the vehicle motion is governed by the dynamic equation, so the behavior of the vehicle has a temporal dependency. Therefore, the interactive LKAS algorithm is designed based on the RNN, which is suitable for modeling temporal dynamic behavior. In addition, since the RNN is a feed-forward neural network, the RNN allows for processing the variable length inputs and reducing the number of parameters, which are the weights and biases of the neural network. These characteristics make the RNN applicable to natural language recognition, such as speech or handwriting [46].

However, if the length of the data is prolonged, gradient vanishing or exploding problems may occur during the learning process. The small gradient for specific features becomes smaller when the depth of the back-propagation is deeper. In contrast, the large gradient becomes larger as it passes through the network layer. To prevent the gradient vanishing and exploding problems, the LSTM cell is applied to the RNN. LSTM cells memorize the activations over arbitrary time intervals [47]. An input, output, and forget gate manages the activations and prevents the vanishing problem. Gated Recurrent Unit (GRU), Vector Autoregressive model (VAR), and ARIMA are frequently used for time series modeling. Since GRU only uses two gates, GRU has fewer parameters than LSTM. Thus, GRU can be trained by using a small set of data. However, if a sufficient dataset is available, it is appropriate to use LSTM. Similarly, it is difficult for VAR to perform long-term prediction on data having a more nonlinear relationship. ARIMA requires stationarity of the time series, which is not an appropriate assumption for vehicle motion modeling. Therefore, RNN with LSTM is used to learn the lane keeping characteristics of the driver.

**Figure 10:**
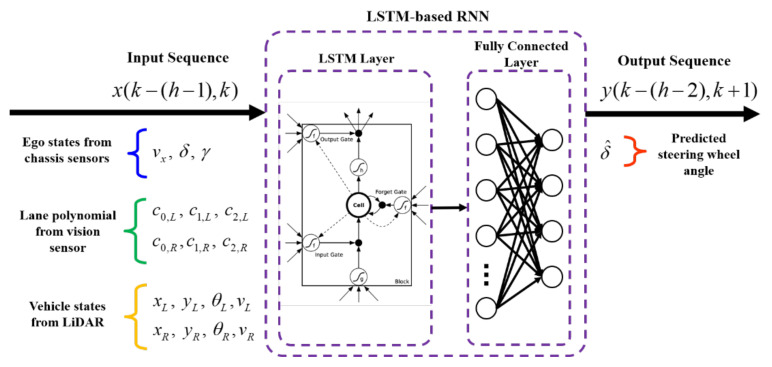
Schematic diagram of the proposed LSTM-RNN-based interactive LKAS model. Adapted from ref. [45].

Figure 10 shows a schematic diagram of the proposed interactive LKAS algorithm. The input and output features with hyperparameters of LSTM-RNN are given in Figure 10. The input sequence consists of ego vehicle states, lane polynomials, and target vehicle states; *k* and *h* are the time index and observation horizon of the RNN. Therefore, *x*(*k* − (*h* − 1), *k*) means the accumulated input features from *k* − (*h* − 1) to *k*-th time index. The proposed model uses the input sequence, *x*(*k* − (*h* − 1), *k*) to predict the output sequence *y*(*k* − (*h* − 2), *k* + 1). The last value of the output sequence is used to control the vehicle to follow the lane. In this study, 20 steps with a sampling time of 100 ms are used to define the input sequences. In other words, the length of the observation is 2 s.

**Figure 11:**
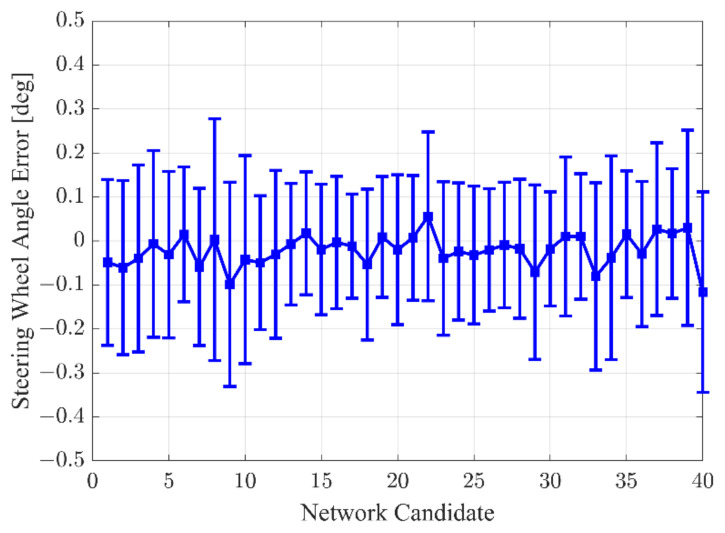
Comparison of the steering wheel angle prediction for the hyperparameter decision.

**Figure 12:**
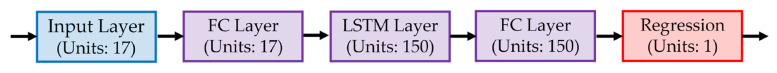
The network configuration with the number of hidden units.

The hyperparameter of the proposed algorithm is determined by comparing the accuracy of the candidate networks. In this study, 40 network candidates are used to find the optimal hyperparameter for the interactive LKAS algorithm. Various combinations of the Fully Connected (FC) layer and LSTM layer are used to define the network candidates. In addition, the number of hidden units of each layer also varies. All network candidates were trained by the same training data samples. The evaluation results of SWA prediction are summarized in Figure 11. Figure 11 shows the error bar of the SWA. The network candidates are sorted according to the number of weights and biases. In other words, candidate #1 uses the smallest number of parameters, and candidate #40 uses the most parameters. Candidate #17 has the smallest standard deviation and a mean close to zero. Therefore, candidate #17 is used to configure the interactive LKAS algorithm, and the architecture of the selected network is depicted in Figure 12.

### 4.3. Network Training

The proposed neural network with an observation horizon of 20 steps was trained and validated by using training and validation data samples. Generally, SGDM (Stochastic Gradient Descent with Momentum) [48], RMSProp, and Adam [49] are representative methods to train neural networks. These methods belong to the stochastic gradient descent method, which replaces the actual gradient with an estimated one. The gradient estimate is calculated from a mini-batch of the training data sample. The mini-batch is randomly selected from the entire data sample. Even if the stochastic gradient descent method is used, there is a possibility of oscillation during the training process. To reduce the oscillation, a momentum term is introduced to the parameter update. In addition, RMSProp automatically adjusts the learning rates for each parameter to improve the training performance. Furthermore, Adam considers the parameter gradients with squared values of gradients to prevent oscillation. In this study, Adam is used to train the proposed neural network. For the training parameters of Adam, the gradient decay factor and the squared gradient decay factor are set as 0.9 and 0.999. An initial learning rate, a learning rate drop factor, and a learning rate drop period are set as 0.005, 0.2, and 125, respectively. A batch size of 256 is used to define the training configuration.

## 5. Results

The proposed interactive LKAS was evaluated by the simulation study based on the collected driving data to analyze the similarity of the SWA decision with human drivers. To show the effectiveness of the proposed algorithm, three base algorithms are used. Base #1 is the integrated algorithm of lane center estimation and path tracking. The lane center estimator is designed based on the extended Kalman filter. The details of the lane center estimator are described in [50]. After estimating the center of the driving lane, a path tracker is used to follow the lane center. The path tracker is designed based on a model-free approach, which does not utilize the vehicle model. The model-free path tracker decided the desired yaw rate by using the relative position between the lane center and the ego vehicle. The steering input is determined to generate the desired yaw rate. The details of the model-free path tracker are described in [51].

The LSTM-based RNN is used to configure Base #2. Unlike the proposed interactive LKAS algorithm, Base #2 used only lane information and states of the ego vehicle for learning. In other words, Base #2 used the same information as conventional LKAS. However, the data-based approach is introduced to consider the characteristics of the driver. Base #3 was designed based on GRU, which replaces the LSTM of the proposed algorithm. The same training, validation, and test data samples are used for Base #2 and Base #3.

### 5.1. Statistical Analysis

**Figure 13:**
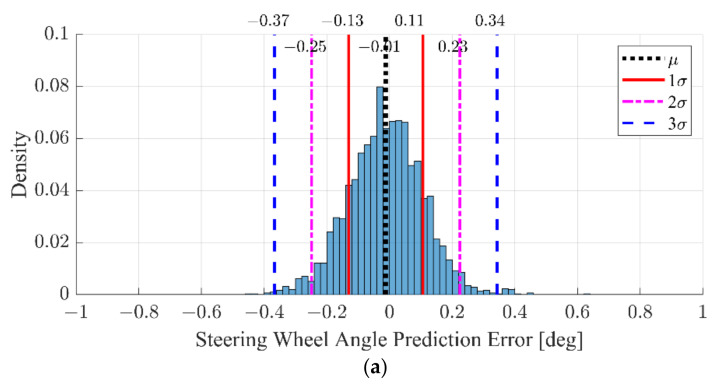

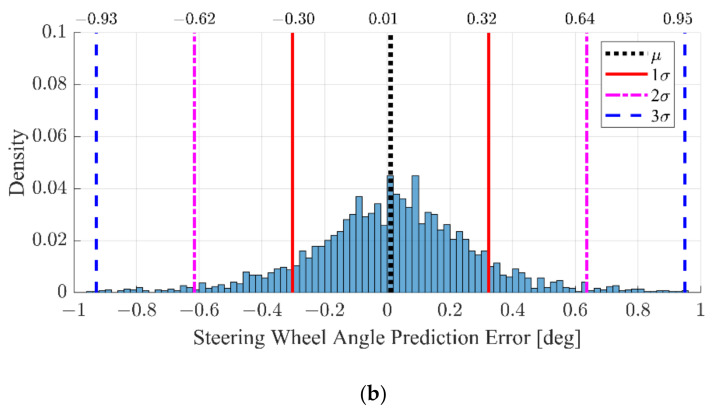
Error histogram: (**a**) interactive lane keeping model and (**b**) conventional lane keeping model, Base #2.

The prediction error of the SWA is summarized in Figure 13. The error of Figure 13 is evaluated using the test data samples, which are not used for the training of the proposed LKAS algorithm. As shown in Figure 13a the mean and standard deviation of the SWA prediction error is −0.01 and 0.12 deg, respectively. The Root Mean Square Error (RMSE) is 0.1189 deg. In addition, the distribution is similar to the normal distribution. This means that the proposed algorithm learns the different responses to left and right targets. Additionally, Figure 13b shows the error histogram of the conventional approaches, Base #2, which only considers the lane information and the ego vehicle states. As shown in Figure 13b, the error distribution of Base #2 is also unbiased because the number of data samples with left and right-lane targets was similarly adjusted. However, the standard deviation is 0.31, which is almost triple from 0.12 of the proposed LKAS algorithm. This is because Base #2 cannot reflect the effect of the surrounding vehicle on SWA decisions. In addition, the RMSE of the Base #2 is 0.2626 deg, which is more than twice that of the proposed algorithm. Therefore, the proposed algorithm reflects the driver’s characteristics into LKAS and shows more precise prediction results than conventional approaches.

### 5.2. Driving Data-Based Simulation

**Figure 14:**
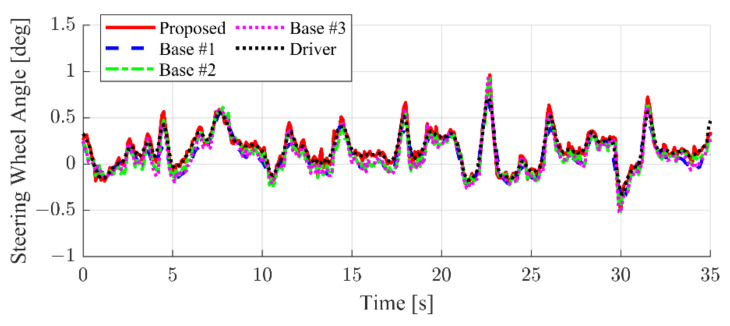
Simulation result comparison when there is no target vehicle around the ego vehicle.

**Figure 15:**
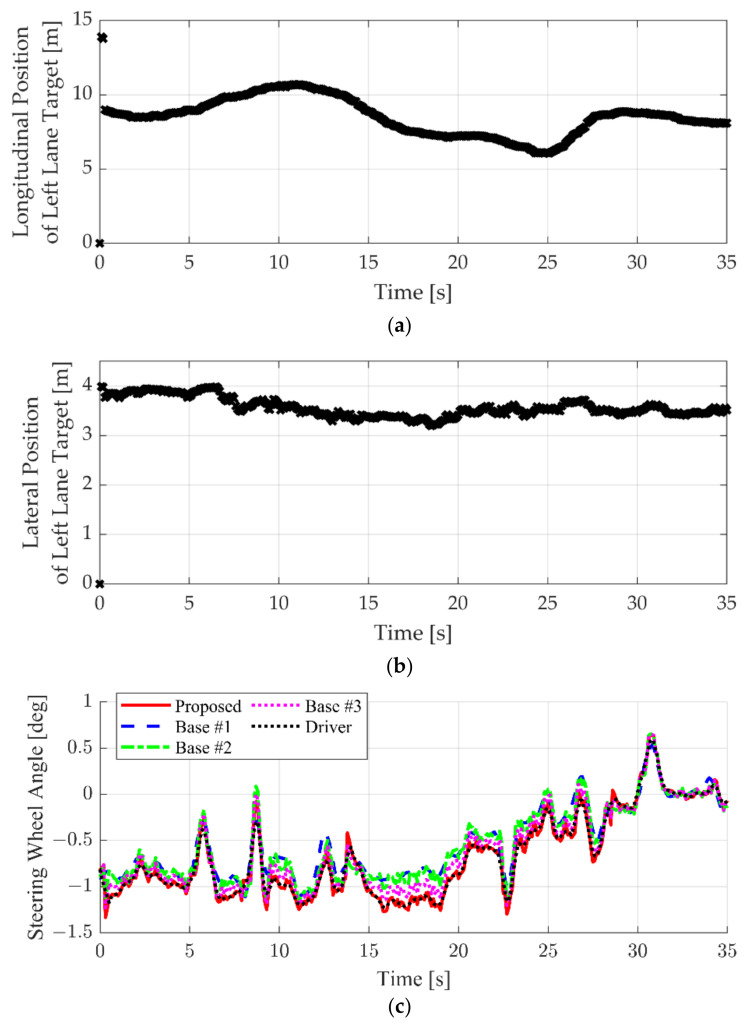
Simulation result comparison when left-lane target exists: (**a**) longitudinal position of the left-lane target; (**b**) lateral position of the left-lane target; and (**c**) steering wheel angle.

**Figure 16:**
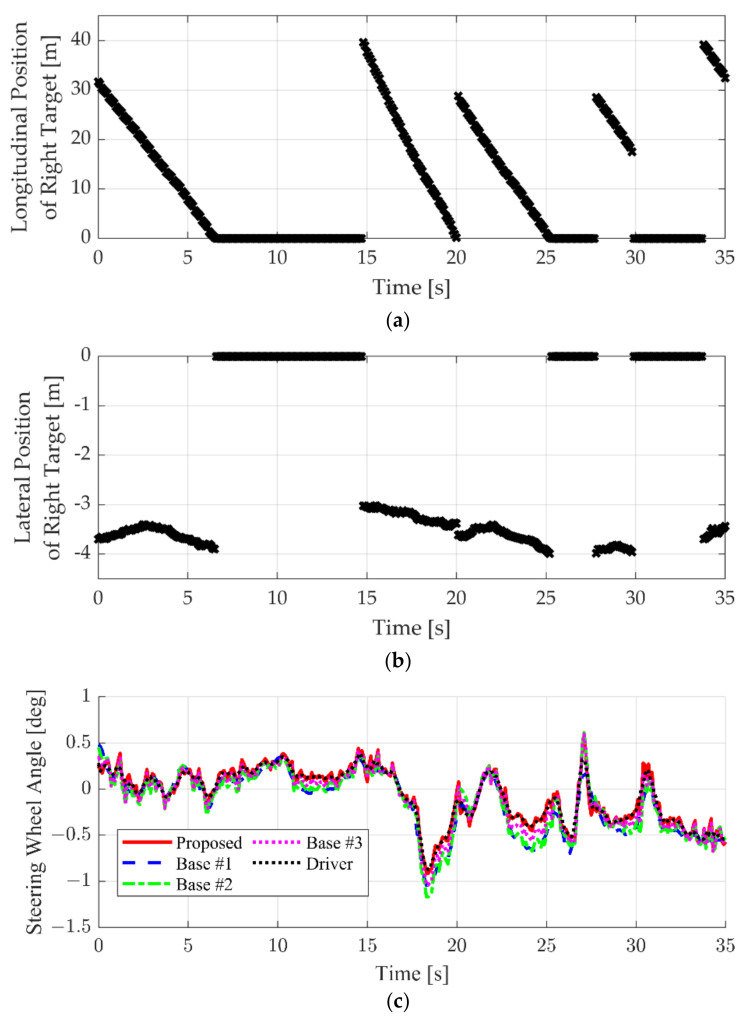
Simulation result comparison when right-lane target exists: (**a**) longitudinal position of the right-lane target; (**b**) lateral position of the right-lane target; and (**c**) steering wheel angle.

**Figure 17:**
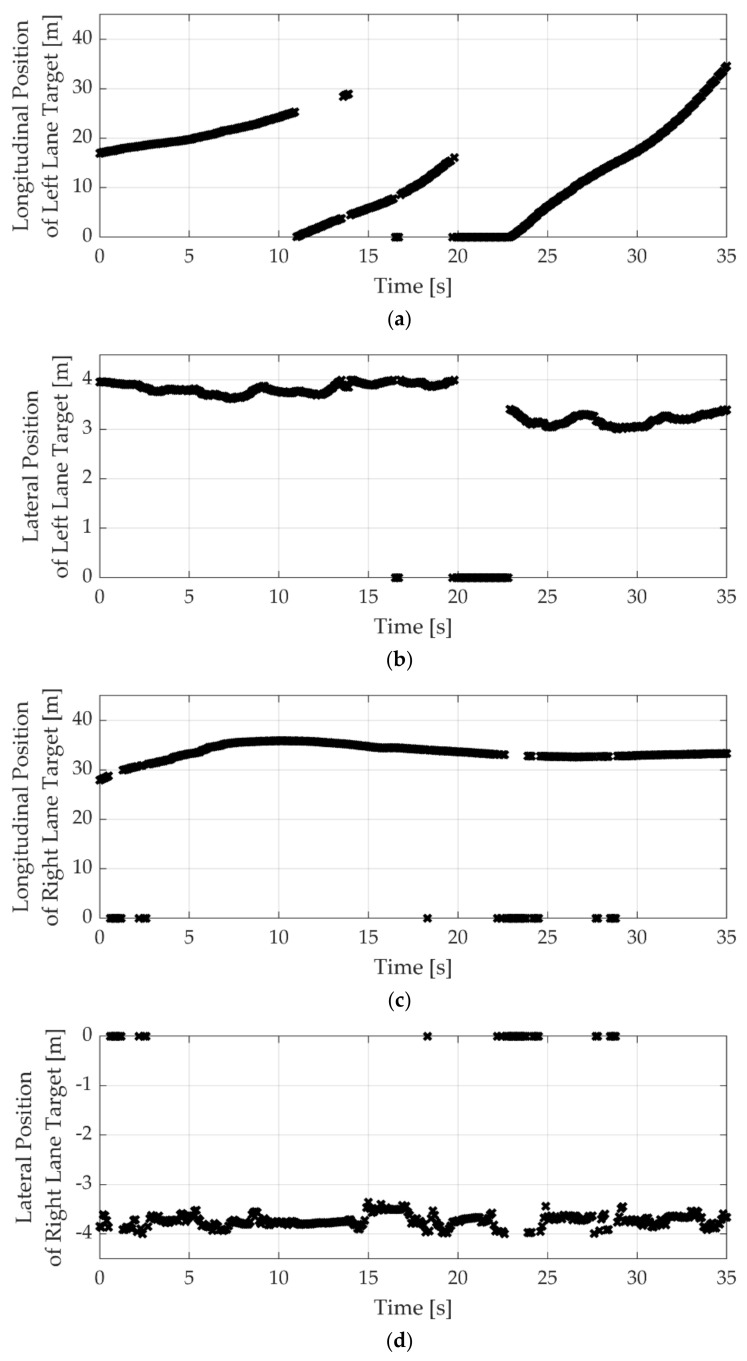

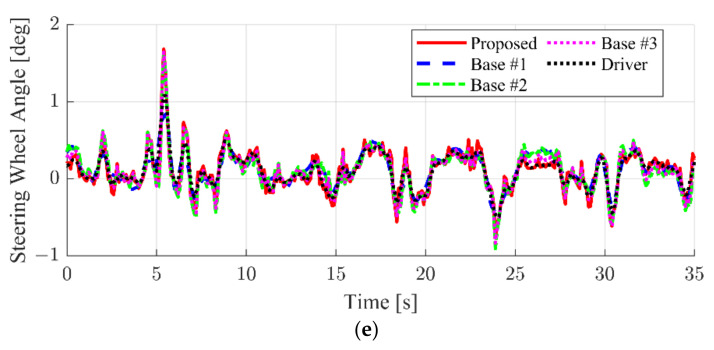
Simulation result comparison when both lane targets exist: (**a**) longitudinal position of the left-lane target; (**b**) lateral position of the left-lane target; (**c**) longitudinal position of the right-lane target; (**d**) lateral position of the right-lane target; and (**e**) steering wheel angle.

The results for the simulation case of no target vehicle, and left, right, and both lane targets are summarized in Figure 14, Figure 15, Figure 16 and Figure 17. The results shown in this section are representative cases selected from the test data samples, which means that the unseen data were used to evaluate the proposed algorithm. First, Figure 14 shows the simulation results of the simple lane keeping scenarios. This means that there is no target vehicle, which can affect the lane keeping of the ego vehicle. As shown in Figure 14, the SWA of the proposed interactive LKAS algorithm is depicted as a red solid line. A blue dashed line, a green dash–dot line, and a magenta dotted line show the results of Base #1, #2, and #3, respectively. The recorded SWA of the human driver is depicted as a black dotted line. Since the lane markers and vehicle states are the only consideration of the SWA decision, the driver, proposed, Base #1, #2, and #3 show similar results. In other words, the extended input features for the proposed algorithm did not cause other problems such as performance degradation in the simple lane keeping scenarios.

The simulation results of the left-lane target case are shown in Figure 15. Figure 15a,b show the longitudinal and lateral position of the left-lane target vehicle with respect to the LCS of the ego vehicle. The left-lane target drove close to the ego vehicle, so the longitudinal position was maintained within about 10 m. Since the origin of the LCS is located at the center of the rear axle, the actual longitudinal clearance with the left-lane target is within 8 m. In this case, the left-lane target gradually approaches the driver’s blind spot, which makes the driver feel anxious. In particular, the relative position of the left-lane target is quietly small, between 15 to 25 s. Since the road curves to the right, more steering input is required to bias the ego vehicle toward the right-lane markers. At this moment, the driver applied more steering inputs to maintain the lateral clearance with the left-lane target. In contrast, since Base #1 is designed to follow the lane center, additional steering inputs are not generated. Therefore, the steering input of base #1 rarely matches the driver’s driving history. This phenomenon also occurred in the results of Base #2. Although Base #2 is an LKAS algorithm constructed by learning based on the same driving data as the proposed algorithm, surrounding vehicles are not considered in determining the steering angle. Therefore, Base #2 shows similar results to Base #1. Base #3 shows more similar results than Base #1 and #2. However, the similarity with the driver of Base #3 is lower than that of the proposed algorithm due to the limitation of the GRU.

The next simulation result considered a situation in which the right-lane targets were overtaken by the ego vehicle. This situation occurs when the speed of the lane in which the ego vehicle drives is high, but congestion occurs in the right lane. The simulation results are summarized in Figure 16. In this case, four right-lane targets were detected. Therefore, four discrete trajectories are shown in Figure 16a,b. In this case, the road curves slightly to the right between 17 to 21 s. The steering input was applied to follow the right curving road. The driver uses less steering input to secure clearance with the vehicle in the slow right lane. The proposed algorithm learned this characteristic and generated an SWA input similar to that of the driver, as shown in Figure 16c. In addition, as given in Figure 16b, when the lateral distance from the right-lane target is close, the effect of the surrounding vehicle is large, which is similar to that of the driver. In contrast, Base #1 and Base #2 tried to follow the lane center and generated larger steering input. Base #3 reflected the influence of surrounding vehicles, but showed lower performance than the proposed algorithm.

Finally, the simulation results with both lane targets are summarized in Figure 17. Before 20 s, the lateral position of the left and right-lane target is about 4 m, as shown in Figure 17b,d. This lateral position is enough lateral clearance to pass by. Even though the longitudinal position is different, as shown in Figure 17a,c, the SWA of the driver and the interactive LKAS algorithms were almost the same, as given in Figure 17e. However, the lateral position of the third left target is about 3 m. Since the nominal width of the passenger vehicle is 1.8 m, the lateral clearance is less than 1.2 m. After 25 s, the driver and proposed algorithm consider the left-lane target and reduce the steering input to increase the lateral clearance. On the contrary, the base algorithms showed the same tendency as in other cases. Therefore, the proposed algorithm provides similar steering inputs to the drivers, who consider the surrounding targets to determine the SWA.

## 6. Conclusions

An interactive lane keeping algorithm is designed by applying a Long Short-Term Memory (LSTM)-based Recurrent Neural Network (RNN) and evaluated through a simulation study using collected driving data. The proposed algorithm considered the lane measurements, ego, and target vehicle states to decide the desired steering wheel angle to reflect the characteristics of the driver. The driving data were collected by the front camera, LiDAR, and chassis sensors. After processing the collected driving data, 46,355 data samples were generated. The proposed algorithm used 32,449; 9281; and 4635 data samples for training, validation, and testing. The statistical analysis results revealed that the steering decision considering the surrounding targets showed results most similar to that of the driver. The mean, standard deviation, and root mean square of the prediction error were −0.01, 0.12, and 0.1189 deg, respectively. Through the case study, the proposed algorithm showed improved lane keeping for each of three cases: no surrounding vehicle, a vehicle on the left, and a vehicle on the right.

Future work on the lane keeping system can be summarized in three aspects: (1) The first is to expand the number of surrounding vehicles to be considered. This study considers the closest two targets on the front left and right. If the increase in computation time is regulated, there is a possibility to improve the lane keeping performance. (2) The second is integration with longitudinal control. The proposed algorithm considers the history of the ego vehicle. In other words, the intention of the longitudinal motion is reflected to determine the steering angle. Recently, the ADAS for longitudinal motion is equipped with lateral motion assistance. Therefore, not only the history of longitudinal behavior but also future inputs can be considered in the LKAS module. (3) Finally, the performance of the proposed learning-based approach can be improved by integration with attention mechanism, convolutional neural network, or reinforcement learning.

## Data Availability

Not applicable.
